# Clinicopathological Features of Intrahepatic Cholangiocarcinoma with Assessment of IDH1 Mutation: A Two-Center Retrospective Study

**DOI:** 10.3390/jcm15135290

**Published:** 2026-07-07

**Authors:** Muge Buyukaksoy, Selin Akturk Esen, Nesrin Turhan, Enes Seyda Sahiner, Imdat Eroglu, Aysenur Sert, Ugur Ozberk, Denizcan Hasturk, Oznur Bal, Efnan Algin, Sebnem Yucel, Nuriye Ozdemir, Onur Ertunc, Dogan Uncu

**Affiliations:** 1 Department of Internal Medicine, University of Health Sciences, Ankara Bilkent City Hospital, 06800 Ankara, Türkiye; enessahiner@hotmail.com (E.S.S.); denizcanh@hotmail.com (D.H.); 2 Department of Medical Oncology, Doruk Nilüfer Hospital, 16110 Bursa, Türkiye; drselin16@hotmail.com; 3 Department of Pathology, University of Health Sciences, Ankara Bilkent City Hospital, 06800 Ankara, Türkiye; nesrinturhan@hotmail.com; 4 Department of Medical Oncology, Faculty of Medicine, Gazi University, 06560 Ankara, Türkiye; 5 Department of Pathology, Faculty of Medicine, Gazi University, 06560 Ankara, Türkiye; 6 Department of Medical Oncology, University of Health Sciences, Ankara Bilkent City Hospital, 06800 Ankara, Türkiye; mdugurozberk@gmail.com (U.O.); doganuncu@yahoo.com (D.U.)

**Keywords:** intrahepatic cholangiocarcinoma, isocitrate dehydrogenase 1 (IDH1) mutation, ALBI score, survival

## Abstract

**Background/Objective**: Intrahepatic cholangiocarcinoma (iCCA) is a highly aggressive malignancy with limited therapeutic options and poor survival outcomes. Recent advances in molecular oncology have highlighted the significance of isocitrate dehydrogenase 1 (IDH1) mutations as potential therapeutic targets. However, data on the prevalence and prognostic implications of IDH1 mutations in patients with iCCA are scarce. This study aimed to retrospectively investigate the IDH1 mutation rate, histopathological features, and survival outcomes of patients with iCCA. **Methods**: We retrospectively analyzed 88 patients diagnosed and treated between 2005 and 2025 at two tertiary oncology centers. Clinical, pathological, and survival data were obtained from the institutional oncology archives and national population databases. IDH1 mutation status was evaluated using polymerase chain reaction and next-generation sequencing of paraffin-embedded tissue samples. **Results**: A total of 88 patients with iCCA were included, with a median age of 62 years, and 62.5% were male. Abdominal pain was the most common presenting symptom (67%). The most frequent stages at diagnosis were stage IB and stage IIIB (22.7% each). Histopathologically, the small-duct subtype (55.7%), nodular morphology (67.0%), and solitary tumors (79.5%) predominated. IDH1 mutation was detected in 2.3% of patients. Curative surgery was performed in 78.2% of cases and was more common in early-stage disease. Gemcitabine–capecitabine was the most frequently used adjuvant regimen, whereas gemcitabine–cisplatin was the most common palliative treatment regimen. Median overall survival differed significantly by albumin-bilirubin (ALBI) grade, with 40.1, 11.1, and 4.4 months for grades 1, 2, and 3, respectively (*p* < 0.001). **Conclusions**: In this multicenter cohort, iCCA was characterized by diverse clinicopathological features and treatment approaches. Surgical resection remains the main treatment modality for patients with localized disease, whereas systemic therapies are more frequently used in advanced stages. The findings highlight the prognostic relevance of baseline clinical and biochemical characteristics and may improve risk stratification in patients with iCCA.

## 1. Introduction

Cholangiocarcinoma (CCA) is an aggressive epithelial cancer arising from the bile ducts. Intrahepatic CCA (iCCA) ranks as the second most common primary liver cancer after hepatocellular carcinoma and represents approximately 3% of all gastrointestinal malignancies. The incidence of iCCA is increasing, and its prognosis remains poor owing to the absence of distinct symptoms, often resulting in diagnosis at advanced stages. The 5-year overall survival rate for iCCA is generally low, ranging between 5% and 20%. Molecular profiling has identified potentially targetable genomic alterations in approximately 40% of patients with CCA. Among these, mutations in the metabolic enzyme isocitrate dehydrogenase 1 (IDH1) are notable, occurring in approximately 14% of iCCA cases but rarely in extrahepatic CCA. Clinically, the prognostic implications of IDH1 mutations remain debated. Some studies have demonstrated long-term progression-free survival in mutation carriers [1], while other studies of advanced disease have reported a negative impact of IDH1 on survival after progression [2]. One study found that IDH1/2 mutations in iCCA were associated with longer overall survival and a longer time to recurrence after resection [3]. Taken together, these findings show the need for integrative analyses combining mutation status and histopathological features to decipher the prognostic value of IDH1 in iCCA. This study aims to retrospectively analyze pathological tissue samples from iCCA patients treated at two centers in Türkiye. The primary objectives are to determine the prevalence of IDH1 mutations and evaluate the effects of other clinicopathological features on survival. The results are expected to provide important insights that can guide future clinical decisions and improve outcomes for patients with this challenging form of cancer.

## 2. Materials and Methods

### 2.1. Study Design

This retrospective, cross-sectional study was conducted at two tertiary centers—the University of Health Sciences Ankara Bilkent City Hospital and Gazi University Faculty of Medicine Hospital—between 1 October 2023 and 1 February 2025. The study included patients who were diagnosed with iCCA between 2005 and 2025. Data for these patients were obtained from the archives of the Medical Oncology Departments at both Ankara Bilkent City Hospital and Gazi University’s Faculty of Medicine.

### 2.2. Population and Participants

Eighty-eight patients with histologically confirmed iCCA were enrolled. Eligible participants were men or women aged 18 years or older. All were under follow-up with a confirmed iCCA diagnosis. Pathology blocks and clinical data from oncology archives had to be accessible. The study aimed for a homogeneous population. Patients with perihilar or distal cholangiocarcinoma, combined or secondary hepatic malignancies (including mixed hepatocellular–cholangiocarcinoma and liver metastases), and extrahepatic malignancies diagnosed in the past five years were excluded. Patients lacking full clinical documentation, staging, follow-up data due to external referrals, or sufficient FFPE tissue were also excluded.

### 2.3. Data Collection

Clinical, laboratory, and survival data were retrospectively retrieved from the medical records of the Department of Medical Oncology of Ankara Bilkent City Hospital and Gazi University Faculty of Medicine Hospital. Histopathological prognostic factors were assessed by experienced pathologists at both participating institutions. Of the 88 patients included, all had complete staging information and reliable follow-up data available. During the follow-up period, 62 patients experienced the event of death. Survival data were obtained from the Turkish National Population Registry.

### 2.4. Laboratory Analysis

Formalin-fixed paraffin-embedded tumor blocks obtained by Tru-Cut biopsy or surgical resection were retrieved from the pathology archives for IDH1 mutation testing. Histological sections (3–5 µm thick) were prepared and examined under a light microscope. DNA extraction was performed using the QIAamp DNA FFPE Tissue Kit (Qiagen Inc., Germantown, MD, USA) according to the manufacturer’s protocol, with the isolates stored at −20 °C until polymerase chain reaction (PCR) amplification.

Primers targeting exon 4 of the IDH1 gene (NM_05896) encompassed codon 132 and amplified from codon 54 to the exon’s C terminus. PCR products were pooled in equal ratios according to yield. DNA quantification was performed using a Nanodrop N1000 spectrophotometer (Thermo Fisher Scientific, Waltham, MA, USA), and the concentration was adjusted to 0.2 ng/µL.

Library preparation for NGS began with DNA fragmentation and adaptor tagging using the Nextera XT kit (Illumina, San Diego, CA, USA). The prepared libraries were sequenced on the Illumina MiSeq platform. Reads were aligned with MiSeq Reporter, and mutation analysis was performed with IGV 2.9.5 (Broad Institute, Cambridge, MA, USA). To detect low-frequency somatic mutations, the allele fraction threshold was set to 0.03, and only genomic regions with read depth ≥ 200× were analyzed. Codon 132, mapped to chr2:208248387–208248389 (hg38), results in an R132 variant if mutated. Each sequencing result was independently reviewed by two molecular biologists to confirm mutation status.

Eligible patients were required to have adequate FFPE tissue samples and complete clinical follow-up data. Patients with insufficient molecular material or failing sequencing quality criteria were excluded from molecular analyses; however, they were retained in the primary clinical cohort due to histologically confirmed disease, complete staging, and reliable follow-up to preserve statistical power for overall survival analyses.

The total number of deaths during the follow-up period was 62 of 88 patients. The specific formula used for the albumin–bilirubin (ALBI) score was also applied in this study as follows: ALBI = (log_10_ total bilirubin × 0.66) + (albumin × −0.085).

### 2.5. Survival Analysis

Progression-free survival (PFS) was defined as the interval from the initiation of first-line chemotherapy to radiologically confirmed disease progression or death from any cause. Overall survival (OS) was defined as the interval from histopathological diagnosis to death from any cause or the last follow-up.

### 2.6. Ethical Approval

This study was approved by the Institutional Ethics Committee of Ankara Bilkent City Hospital (protocol number E1-23-3976, dated 6 September 2023) and conducted in accordance with good clinical practice, applicable laws, and the Declaration of Helsinki.

According to the decision of Institutional Ethics Committee of Ankara Bilkent City Hospital, informed consent was not required due to the retrospective study design and the use of anonymized data, which prevented participant identification.

### 2.7. Statistical Analyses

Statistical analyses were performed using IBM SPSS Statistics for Windows, version 26.0 (IBM Corp., Armonk, NY, USA). The Kolmogorov–Smirnov test was used to check data normality. Continuous variables are shown as median, minimum, and maximum values. Categorical variables are shown as counts and percentages. The Kaplan–Meier method was used for survival analysis. Survival curves were compared with the log-rank test. Results with *p* < 0.05 were statistically significant.

## 3. Results

This study included 88 patients with iCCA who were followed at two centers. The median age at diagnosis was 62 years (range, 31–79). The study group consisted of 55 men (62.5%) and 33 women (37.5%), reflecting male predominance.

Stages IB and IIIB were the most frequent, each accounting for 22.7% (n = 15) of cases.

In the evaluation of distant metastasis sites, liver and nonregional lymph node metastases were detected at equal frequencies, each observed in 5.7% (n = 4) of patients.

Table 1 presents the baseline demographic and clinicopathological characteristics of patients with iCCA.

PCR-based analysis revealed an IDH1 wild-type profile in 85.2% (n = 75) of patients and an IDH1 mutation in 2.3% (n = 2).

Furthermore, the mutation status was indeterminate in 12.5% (n = 11) of patients due to inadequate tumor tissue.

**Figure 1:**
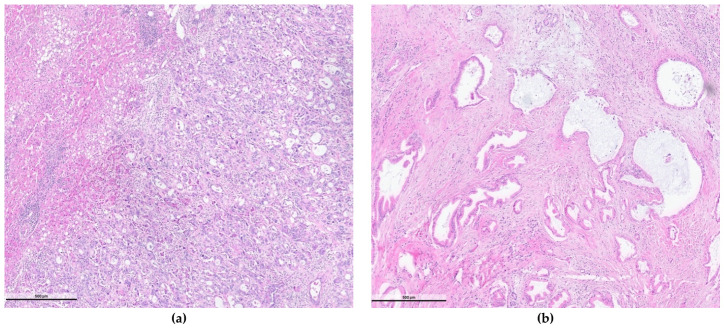
Representative histopathological features of the intrahepatic cholangiocarcinoma subtypes. (**a**) Large duct-type iCCA demonstrating large irregular glandular structures with abundant mucin production, resembling intrahepatic bile duct morphology, set within a desmoplastic stroma. (**b**) Small duct-type iCCA is characterized by small angulated tubular structures with a high nuclear-to-cytoplasmic ratio and scant mucin embedded in a densely fibrotic stroma. (Hematoxylin and eosin staining, scale bar = 500 µm).

With respect to histopathological subtype, the small-duct type was the most prevalent, observed in 55.7% (n = 49) of cases (Figure 1). Morphologically, the nodular form was the most frequent pattern, identified in 67% (n = 59) of the patients. Additionally, regarding tumor growth patterns, infiltrative growth was observed in 45.5% (n = 40) of the cases. Solitary tumors were present in 79.5% (n = 70) of patients.

Moving from tumors to the surrounding tissue, histopathological assessment of the nontumorous liver parenchyma demonstrated that hepatocellular cholestasis (19.3%, n = 17) and mild portal fibrosis with inflammation (17%, n = 15) were the most frequently observed lesions.

Regarding tumor grade, grade 2 differentiation was the most frequent, identified in 70.5% (n = 62) of patients. Additionally, capsular invasion was absent in 68.2% (n = 60) of patients, and adjacent structure invasion was not observed in 65.9% (n = 58) of patients.

Grade 1 tumor budding was the most common, accounting for 45.5% (n = 40) of the cases.

Lymphovascular and perineural invasions were detected in 55.7% (n = 49) and 47.7% (n = 42) of patients, respectively, and microvascular invasion was detected in 9.1% (n = 8).

In addition to these findings, assessment of the surgical margins demonstrated that negative margins were achieved in 58% (n = 51) of the cases. Grade 1 ALBI score was the most frequent classification, observed in 39.8% (n = 35) of patients.

Collectively, the pathological, molecular, and biochemical characteristics of the patients diagnosed with iCCA are presented in Table 2.

Eighty-eight patients were included in the study. One patient was lost to follow-up after enrollment and excluded from analyses requiring treatment intent, making treatment intent evaluable in 87 patients. Of these, 68 (78.2%) underwent curative surgery, while 19 (21.8%) received a palliative or nonsurgical approach. Surgical information was unavailable for 14 patients. As shown in Table 3, most patients undergoing curative surgery were diagnosed at an early disease stage: 41.2% (n = 28) had stage I, 19.1% (n = 13) stage II, 30.9% (n = 21) stage III, and 1.5% (n = 1) stage IV. Among those not undergoing curative surgery, the majority, 68.4% (n = 13), had stage IV disease.

Treatment analysis showed 42.6% (n = 29) of patients who had curative surgery received adjuvant chemotherapy. Among these patients, 10.3% (n = 7) also received radiotherapy, and 13.2% (n = 9) received adjuvant chemoradiotherapy. In the nonsurgical group, 63.2% (n = 12) received palliative chemotherapy for metastatic disease (Table 3).

For adjuvant chemotherapy, gemcitabine–capecitabine was most common (32.4%, n = 22), followed by capecitabine alone (17.6%, n = 12). In palliative chemotherapy, gemcitabine–cisplatin was used most often (47.4%, n = 9) (Table 3).

**Figure 2:**
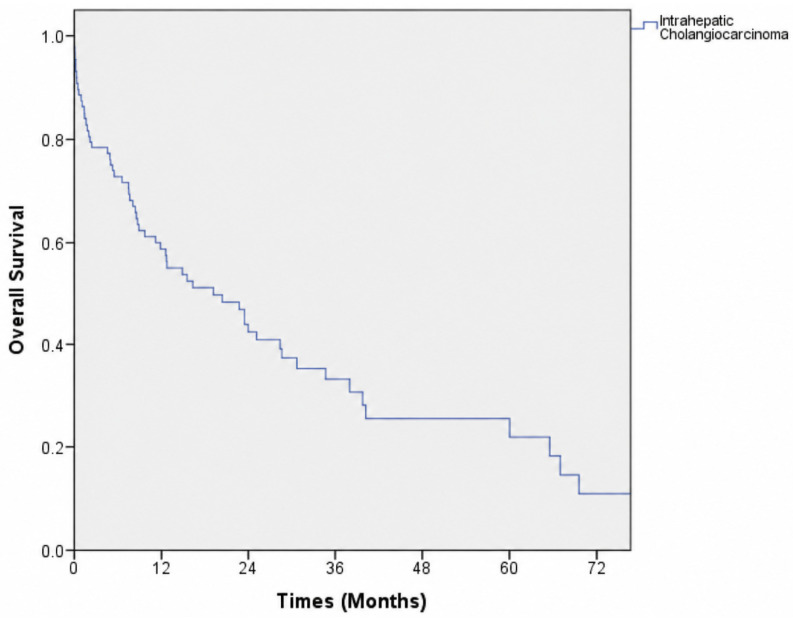
Kaplan–Meier curve for median overall survival in patients with intrahepatic cholangiocarcinoma.

The median OS of all analyzed patients was 19.1 months (95% CI: 9.7–28.5) (Figure 2).

**Figure 3:**
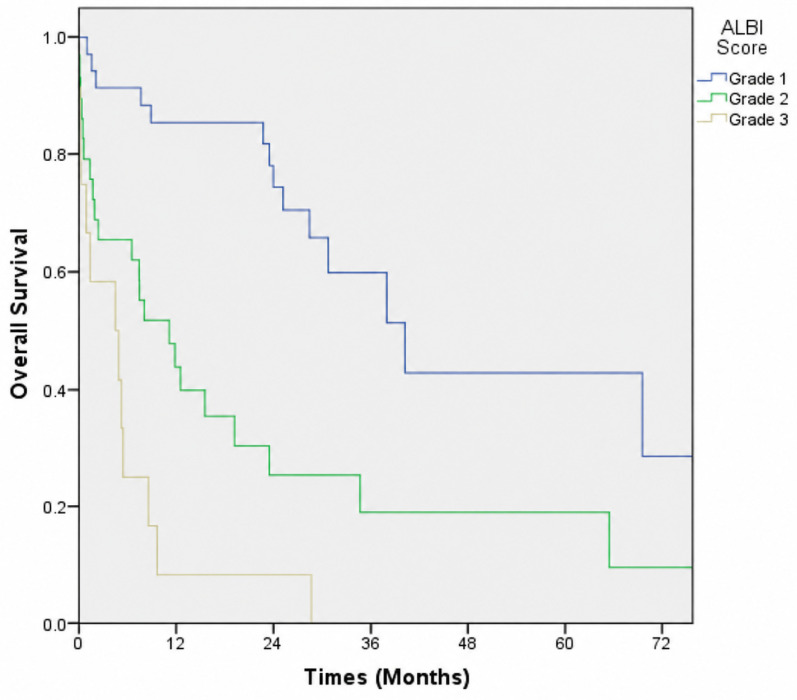
Kaplan–Meier curves for median overall survival according to albumin–bilirubin (ALBI) scores in patients with intrahepatic cholangiocarcinoma.

Stratification by ALBI score revealed striking differences in OS, with rates of 40.1 months (95% CI: 26.6–53.7) for grade 1, 11.1 months (95% CI: 4.1–18.1) for grade 2, and 4.4 months (95% CI: 0.02–10.4) for grade 3 (*p* < 0.001). Pairwise comparisons confirmed significant survival differences across all grades (grade 1 vs. 2, *p* < 0.001; grade 1 vs. 3, *p* < 0.001; grade 2 vs. 3, *p* = 0.017) (Figure 3).

## 4. Discussion

Although iCCA is a rare and aggressive gastrointestinal malignancy, its incidence has been observed to increase over the past decade [4]. In the treatment of malignancies, detailed analyses of the tumor’s genetic profile and evaluation of prognostic factors are becoming increasingly important in designing targeted and individualized therapeutic strategies. Recent advances in genomic profiling have enabled the identification of targetable alterations such as variations in IDH1, FGFR2, BRAF V600E, HER2, and NTRK [5], leading to the integration of NGS-based panels into clinical practice, as recommended by the European Society for Medical Oncology, for detecting ESCAT tier I alterations including IDH1 mutations, FGFR2 and NTRK fusions, and MSI-high status [6]. In our study, IDH1 mutation was retrospectively analyzed using DNA extracted from pathological tissue samples, with a mutation rate of 2.3%. While pooled analyses from the literature indicate an overall prevalence of approximately 13.1% in iCCA, a recent multinational review by Skok et al. [7], reports that the molecular frequency of IDH1 mutations in iCCA exhibits a remarkably wide spectrum, ranging from 1% to 18% (specifically 8–18% in intrahepatic subtypes), heavily influenced by geographic and institutional variations. The lower frequency observed in our study may reflect ethnic differences, as well as technical limitations, since IDH1 testing could not be performed in 11 patients (12.5%) in our study. Moreover, only tissue blocks were analyzed; liquid biopsy might provide different detection rates than tissue biopsy.

However, because only two IDH1-mutant cases were identified in our study, a meaningful statistical evaluation of the association between IDH1 status and overall survival was not feasible. Therefore, our findings should be regarded primarily as descriptive observations of iCCA. Additionally, the marked molecular heterogeneity of iCCA suggests that its biological complexity cannot be fully captured by single-gene analyses alone. Notably, recent reviews across diverse cancer types, including iCCA, have highlighted the importance of integrating genomic, transcriptomic, epigenomic, proteomic, and metabolomic data through multi-omics investigations [8,9]. These findings underscore the potential of integrated molecular profiling to refine prognostic stratification beyond conventional clinicopathological factors.

In this study, we investigated the histopathological characteristics associated with iCCA by analyzing liver biopsy and resection samples. Our findings suggest a multifactorial etiology for iCCA development. We observed histopathological features associated with fibrosis and cirrhosis, as well as MASH, in 12.5% of patients. This points to a potential link among metabolic syndrome, fatty liver disease, and iCCA, a finding consistent with the results of De Lorenzo et al. [10], who reported a MASH prevalence of 22.5% in patients without classic iCCA risk factors. Furthermore, the presence of hepatocellular and hepatocanalicular cholestasis indicates that impaired bile drainage may contribute to carcinogenesis. Other features such as intraductal tubulopapillary neoplasms, chronic cholangitis, and foreign body-type granulomas suggest that premalignant changes, chronic inflammation, and immune responses also contribute to tumor progression.

Analysis of stage distribution revealed that most patients were diagnosed at stage IB or IIIB, indicating locally advanced or advanced disease at presentation. This aligns with the recognized challenge of early iCCA diagnosis, as most cases are identified after symptom onset [6,11,12]. Regarding metastatic patterns, distant metastases were observed in 15.9% of patients, most commonly to the liver, followed by nonregional lymph nodes and bone. These results align with previously reported dissemination patterns [13], confirming that nonregional lymph nodes and bone are frequent sites of extrahepatic spread in iCCA.

In our study, 68 patients underwent curative surgeries. Notably, the proportion of patients who underwent curative resection in our study (68 out of 87, or 78.2%) was significantly higher than the rates of 20–30% reported in previous studies [14]. This observed difference may be attributable to specific institutional selection criteria, comprehensive multidisciplinary evaluation processes, or the unique characteristics of our patient population. However, these findings require confirmation through large-scale studies. The most frequent adjuvant chemotherapy regimens were gemcitabine–capecitabine and capecitabine, whereas gemcitabine–cisplatin and gemcitabine–carboplatin was predominant in palliative settings. According to the literature, the standard first-line systemic therapies for patients with unresectable or advanced iCCA are gemcitabine–cisplatin–durvalumab or pembrolizumab, with FOLFOX recently accepted as the standard second-line treatment [15,16].

We evaluated the ALBI scores in our cohort, with 39.8% of patients classified as grade 1, 33% as grade 2, and 13.6% as grade 3. Survival declined markedly with increasing grade, consistent with previous studies linking higher ALBI grades to a poorer prognosis [17]. Thus, our results further corroborate the observations in the literature.

While some studies have reported a significant impact of perineural invasion on both OS and recurrence-free survival [18], others have found no significant effect in univariate or multivariate analyses [19,20]. Perineural invasion is not recognized as a prognostic factor in the 8th edition of the American Joint Committee on Cancer guidelines or other guidelines [21]. Larger studies and meta-analyses are needed to clarify whether it can be considered an independent prognostic factor.

Tumor necrosis has been linked to poor outcomes in several studies. It is thought to reflect hypoxia-driven tumor aggressiveness. Tsilimigras et al. reported that extensive tumor necrosis is associated with poorer survival outcomes in patients with intrahepatic cholangiocarcinoma [22]. Similarly, infiltrative growth patterns have been tied to an unfavorable prognosis. Current guidelines recognize the periductal-infiltrative subtype of iCCA as an adverse morphological variant [21]. Tumor budding is a well-established prognostic marker in various malignancies, particularly colorectal cancer. In that context, it is associated with increased invasiveness, metastatic potential, and poor survival [23]. Although its prognostic significance in iCCA has been explored, the available evidence is limited. Further studies are needed to clarify its role in this disease.

Our study had several limitations. First, its retrospective design and incomplete clinical data restricted the scope of some analyses. Second, molecular profiling was based solely on FFPE tissue samples, and the absence of cell-free DNA analysis via liquid biopsy may have led to an underestimation of the true IDH1 mutation prevalence. Additional technical factors, including tissue adequacy, DNA extraction efficiency, primer design, and laboratory conditions, could have further constrained the sensitivity of mutation detection. The observed IDH1 mutation rate of 2.3% was notably lower than the 13% reported in larger international studies [7]. While this difference may partly reflect population-specific genetic backgrounds, the small sample size and limited demographic diversity preclude definitive conclusions, and larger prospective studies are needed to clarify the prevalence of IDH1 mutations in Turkish patients with iCCA.

Furthermore, our molecular analysis was limited to targeted sequencing of IDH1 exon 4. The absence of a broader NGS panel and complementary immunohistochemical profiling prevented more comprehensive molecular characterization. Nevertheless, this study lays a foundation for understanding the clinicopathological and molecular characteristics of iCCA in an understudied ethnic group. To the best of our knowledge, it represents the first dedicated IDH1 mutation analysis and comprehensive histopathological assessment of this malignancy in a population currently absent from the literature on iCCA molecular epidemiology.

Due to the small number of patients and the highly heterogeneous patient group, we were unable to perform Cox regression analysis to evaluate prognostic factors.

## 5. Conclusions

In this multicenter cohort, patients with iCCA exhibited considerable clinical, pathological, and treatment-related heterogeneity. Curative surgery was predominantly performed in patients with earlier-stage disease, whereas patients with advanced-stage disease were more likely to receive palliative treatment. The small-duct subtype, nodular morphology, and solitary tumor pattern were the most common pathological features. Survival outcomes varied significantly according to liver functional reserve, as assessed by the ALBI grade, emphasizing the importance of both tumor- and patient-related factors in determining prognosis. These findings provide a comprehensive characterization of iCCA in a real-world population and support the use of readily available clinicopathological and biochemical parameters for prognostic assessment and treatment planning. Further studies are needed to validate these observations and refine the prognostic stratification of patients with iCCA.

## Figures and Tables

**Table 1:** Baseline Demographic and Clinicopathological Characteristics of Patients with Intrahepatic Cholangiocarcinoma.

Variables	Group	*n* (%) 88 (100)
**Age at diagnosis, years**		62 (31–79)
**Tumor** **diameter (mm)**		60 (17–174)
**Sex**	Female	33 (37.5)
	Male	55 (62.5)
**ECOG PS**	0	4 (4.5)
	1	74 (84.1)
	2	7 (8)
	3	3 (3.4)
**Smoking history**	None	43 (48.9)
	Active smoker	12 (13.6)
	Ex-smoker	16 (18.2)
	Unknown	17 (19.3)
**Alcohol consumption**	No	66 (75)
	Yes	5 (5.7)
	Unknown	17 (19.3)
**Family history of CCA**	Absent	71 (80.7)
	Unknown	17 (19.3)
**HBV infection**	Absent	74 (84.1)
	Inactive chronic HBV	6 (6.8)
	Immunoactive, HBeAg-negative	6 (6.8)
	Occult HBV	2 (2.3)
**HCV infection**	Absent	88 (100)
**Cholelithiasis**	Absent	58 (65.9)
	Present	18 (20.5)
	Unknown	12 (13.6)
**Stage**	Stage IA	8 (9.1)
	Stage IB	20 (22.7)
	Stage II	10 (14.8)
	Stage IIIA	13 (3.4)
	Stage IIIB	20 (22.7)
	Stage IV	14 (15.9)
	Unknown	10 (11.4)
**Liver metastasis**	Absent	74 (84.1)
	Present	5 (5.7)
	Unknown	9 (10.2)
**Peritoneal metastasis**	Absent	77 (87.5)
	Present	2 (2.3)
	Unknown	9 (10.2)
**Nonregional lymph node metastasis**	Absent	74 (84.1)
	Present	5 (5.7)
	Unknown	9 (10.2)
**Lung metastasis**	Absent	78 (88.6)
	Present	1 (1.1)
	Unknown	9 (10.2)
**Bone metastasis**	Absent	76 (86.4)
	Present	3 (3.4)
	Unknown	9 (10.2)
**Adrenal metastasis**	Absent	78 (88.6)
	Present	1 (1.1)
	Unknown	9 (10.2)

Note: Age at diagnosis and tumor diameter are presented as median (minimum–maximum); all other variables are expressed as count (percentage). CCA: Cholangiocarcinoma; ECOG PS: Eastern Cooperative Oncology Group performance status; HBV: hepatitis B virus; HCV: hepatitis C virus.

**Table 2:** Pathological, Molecular, and Biochemical Characteristics of Patients with Intrahepatic Cholangiocarcinoma.

Variables		n (%) 88 (100)
**IDH1 mutation**	Negative	75 (85.2)
	Positive	2 (2.3)
	Insufficient material for analysis	11 (12.5)
**Histopathological subtype**	Small-duct type	49 (55.7)
	Large-duct type	27 (30.7)
	Unknown	12 (13.6)
**Morphology**	Nodular	59 (67)
	Sclerosing	11 (12.5)
	Papillary	4 (4.5)
	Unknown	14 (15.9)
**Solitary/multiple**	Solitary	70 (79.5)
	Multiple	8 (9.1)
	Unknown	10 (11.4)
**Nontumorous** **liver parenchyma**	Unremarkable pathology	12 (13.6)
	HBV-related liver cirrhosis	2 (2.3)
	MASH-related liver cirrhosis	1 (1.1)
	Cryptogenic liver cirrhosis	2 (2.3)
	Primary sclerosing cholangitis	2 (2.3)
	MASH	11 (12.5)
	Arising in the background of intraductal tubulopapillary neoplasm	2 (2.3)
	Hepatocellular cholestasis	17 (19.3)
	Incomplete cirrhosis	1 (1.1)
	Hepatocanalicular cholestasis	10 (11.4)
	Mild portal fibrosis and inflammation	15 (17)
	Cholangitis	1 (1.1)
	Foreign body-type granulomatous reaction	2 (2.3)
	Unknown	10 (11.4)
**Grade**	Grade X	10 (11.4)
	Grade 1	15 (17)
	Grade 2	62 (70.5)
	Grade 3	1 (1.1)
**Liver capsule invasion**	Absent	60 (68.2)
	Present	19 (21.6)
	Unknown	9 (10.2)
**Tumor growth** **pattern**	Expansile	36 (40.9)
	Infiltrative	40 (45.5)
	Unknown	12 (13.6)
**Adjacent structure invasion**	Absent	58 (65.9)
	Present	20 (22.7)
	Unknown	10 (11.4)
**Tumor budding**	Absent	6 (6.8)
	Grade 1	40 (45.5)
	Grade 2	13 (14.8)
	Grade 3	16 (18.2)
	Unknown	13 (14.8)
**Necrosis**	Absent	41 (46.6)
	<30%	24 (27.3)
	30–60%	9 (10.2)
	60–90%	1 (1.1)
	Unknown	13 (14.8)
**Lymphovascular invasion**	Absent	27
	Present	49 (55.7)
	Unknown	12 (13.6)
**Perineural invasion**	Absent	33 (37.5)
	Present	42 (47.7)
	Unknown	13 (14.8)
**Microvascular invasion**	Absent	70 (79.5)
	Present	8 (9.1)
	Unknown	10 (11.4)
**Surgical margin**	Negative	51 (58)
	Positive	23 (26.1)
	Unknown	14 (15.9)
**ALBI score**	Grade 1	35 (39.8)
	Grade 2	29 (33)
	Grade 3	12 (13.6)
	Unknown	12 (13.6)
**CA 19-9 (U/mL)**		58.1 (0.8–67,838.6)
**CEA (ng/mL)**		1.7 (0.05–1901)
**AFP (µg/L)**		2.7 (0.05–133.2)
**Biochemical parameters** **at diagnosis**		
**Total bilirubin (mg/dL)**		1.2 (0.2–30.6)
**Direct bilirubin (mg/dL)**		0.4 (0.1–20.9)
**LDH (U/L)**		241 (120–862)
**Albumin (g/dL)**		39.3 (12–50)

AFP: Alpha-fetoprotein; ALBI: albumin–bilirubin score; CA 19-9: carbohydrate antigen 19-9; CEA: carcinoembryonic antigen; IDH1: isocitrate dehydrogenase 1; LDH: lactate dehydrogenase; MASH: metabolic dysfunction-associated steatohepatitis.

**Table 3:** Treatment Regimens in Patients with Intrahepatic Cholangiocarcinoma.

Variables		Curative Surgery	
		Yes	No
		n (%) 68 (100)	n (%) 19 (100)
**Stage**	Stage 1	28 (41.2)	0 (0)
	Stage 2	13 (19.1)	0 (0)
	Stage 3	21 (30.9)	1 (5.3)
	Stage 4	1 (1.5)	13 (68.4)
	Unknown	5 (7.4)	5 (26.3)
**Treatment regimen**	Treatment refusal	2 (2.9)	0 (0)
	Surgery + adjuvant CT	29 (42.6)	0 (0)
	Surgery + adjuvant CT followed by adjuvant RT	7 (10.3)	0 (0)
	Metastatic (palliative) CT	0 (0)	12 (63.2)
	Neoadjuvant CT + surgery + adjuvant CT/RT	2 (2.9)	0 (0)
	Surveillance after surgery without therapy	1 (1.5)	0 (0)
	Patients who died before receiving treatment	14 (20.6)	5 (26.3)
	Unknown	13 (19.1)	2 (10.5)
**Adjuvant CT**	Gemcitabine	2 (2.9)	
	Gemcitabine + capecitabine	22 (32.4)	
	Capecitabine	12 (17.6)	
	Gemcitabine + oxaliplatin	1 (1.5)	
	Unknown	14 (20.6)	
	Treatment refusal	3 (4.4)	
	Patients who died before receiving treatment	14 (20.6)	
**Palliative CT**	Gemcitabine		1 (5.3)
	Gemcitabine + cisplatin		9 (47.4)
	Gemcitabine + carboplatin		1 (5.3)
	Unknown		3 (15.8)
	Patients who died before receiving treatment		5 (26.3)
**CRT**	None	32 (47.1)	12 (63.2)
	Adjuvant	9 (13.2)	0 (0)
	Unknown	13 (19.1)	2 (10.5)
	Patients who died before receiving treatment	14 (20.6)	5 (26.3)

CT: Chemotherapy; CRT: chemoradiotherapy; RT: radiotherapy.

## Data Availability

All data generated or analyzed during this study are included in this published article. The genomic mutation analysis data were deposited in the European Genome-phenome Archive (EGA) under accession number EGAS50000001638 (Direct link: https://ega-archive.org/studies/EGAS50000001638 (accessed on 25 February 2026). Owing to the sensitive nature of human genomic data, these datasets are available under controlled access. Researchers wishing to access the raw data must submit a request to the corresponding Data Access Committee (DAC), as outlined in the EGA repository. Additional inquiries may be directed to the corresponding author (mugebuyukaksoy@gmail.com).
